# The effect of 2-[(aminopropyl)amino] ethanethiol (WR-1065) on radiation induced DNA double strand damage and repair in V79 cells.

**DOI:** 10.1038/bjc.1987.97

**Published:** 1987-05

**Authors:** C. P. Sigdestad, S. H. Treacy, L. A. Knapp, D. J. Grdina

## Abstract

Radiation induced DNA double strand breaks are believed to be important lesions involved in processes related to cell killing, induction of chromosome aberrations and carcinogenesis. This paper reports the effects of the radioprotector 2-[(aminopropyl)amino]ethanethiol (WR-1065) on radiation-induced DNA damage and repair in V79 cells using the neutral elution method performed at pH 7.2 or pH 9.6. WR-1065 (4 mM) was added to the culture medium either 30 minutes prior to and during irradiation with Cobalt-60 gamma rays (for dose response experiments) or during the repair times tested (for DNA rejoining experiments). The results indicate that WR-1065 is an effective protector against the formation of radiation-induced double-strand breaks in DNA as measured using a neutral elution technique at either pH. The protector reduced the strand scission factors by 1.44 and 1.77 in experiments run at pH 9.6 and pH 7.2, respectively. The kinetics of DNA double-strand rejoining were dependent upon the pH at which the neutral elution procedure was performed. Unlike the results obtained with alkaline elution, rejoining of DNA breaks was unaffected by the presence of WR-1065 at either pH.


*Br. J. Cancer* (1987), **55**, 477–482 © The Macmillan Press Ltd., 1987

### The effect of 2-[(aminopropyl)amino] ethanethiol (WR-1065) on radiation induced DNA double strand damage and repair in V79 cells

C.P. Sigdestad[1], S.H. Treacy[2], L.A. Knapp[2] & D.J. Grdina[2,3]

[1]*Radiation Oncology Department, J. Graham Brown Cancer Center, University of Louisville, School of Medicine, 529 S. Jackson St., Louisville, KY 40202,* [2]*Division of Biological and Medical Research, Argonne National Laboratory, Argonne, IL 60439-4833 and* [3]*Radiation Oncology Department, University of Chicago, School of Medicine, Chicago, IL 60637 USA.*

**Summary** Radiation induced DNA double strand breaks are believed to be important lesions involved in processes related to cell killing, induction of chromosome aberrations and carcinogenesis. This paper reports the effects of the radioprotector 2-[(aminopropyl)amino] ethanethiol (WR-1065) on radiation-induced DNA damage and repair in V79 cells using the neutral elution method performed at pH 7.2 or pH 9.6. WR-1065 (4 mM) was added to the culture medium either 30 minutes prior to and during irradiation with Cobalt-60 gamma rays (for dose response experiments) or during the repair times tested (for DNA rejoining experiments). The results indicate that WR-1065 is an effective protector against the formation of radiation-induced double-strand breaks in DNA as measured using a neutral elution technique at either pH. The protector reduced the strand scission factors by 1.44 and 1.77 in experiments run at pH 9.6 and pH 7.2, respectively.

The kinetics of DNA double-strand rejoining were dependent upon the pH at which the neutral elution procedure was performed. Unlike the results obtained with alkaline elution, rejoining of DNA breaks was unaffected by the presence of WR-1065 at either pH.

Considerable interest has recently been expressed in the use of phosphorylthioate drugs as adjuvants to radiotherapy (Kligerman *et al.*, 1980, 1984; Takahasi *et al.*, 1986). This interest is based upon an early observation by Yuhas and Storer, (1969) that WR-2721 differentially protects normal as compared to tumour cells. Utley *et al.*, (1976) using whole-body autoradiography with labelled WR-2721, later demonstrated that little or no uptake of the protector was observed in an EMT/6 tumour, although wide distribution was noted in surrounding normal tissues.

Yuhas (1980) and Yuhas *et al.*, (1980) suggested that these radiation-protective drugs may have a more general role in cancer treatment. This suggestion was confirmed by Milas *et al.* (1984), who reported the anticarcinogenic effect of WR-2721 in irradiated mice. In addition, protectors have been shown to be antimutagenic (Grdina *et al.*, 1985a; Grdina & Nagy, 1986) and antineoplastic (Hill *et al.*, 1986) and to reduce the formation of radiation-induced preneoplastic foci in rats (Grdina *et al.*, 1985*b*).

It is clear that DNA damage and repair mechanisms are involved in these effects. Therefore, it is important to study the interaction of protectors at the subcellular level. Grdina and Nagy (1986) have shown that WR-1065 (the dephosphorylated derivative of WR-2721) protects against the formation of single-strand breaks (SSB) as determined by alkaline elution. In addition, these authors reported that the presence of the protector during repair inhibits strand rejoining in irradiated cells.

Double-strand breaks (DSB) are widely believed to be more biologically relevant than SSB, and DSB have been implicated as the critical event in radiation-induced cell killing (Hutchinson, 1978; Radford, 1985; Ward, 1986). The neutral elution technique, developed by Bradley and Kohn (1979) measures DNA DSB. These observations prompted a series of studies to determine whether the dephosphorylated WR-2721 (WR-1065) protects V79 cells from DSB or modulates repair processes after irradiation. Because some workers have suggested (Tilby *et al.*, 1984; Evans *et al.*, 1986) that DNA elution at pH 9.6 modifies the quality of breaks, we assayed DSB formation both at pH 9.6 and pH 7.2.

Correspondence: D.J. Grdina, BIM/202 Argonne National Laboratory, 9700 S. Cass Avenue, Argonne, IL 60439-4833, USA.
Received 26 September 1986; and in revised form, 11 February 1987.

### Materials and methods

### *Cell preparation*

V79-B310H Chinese hamster cells were cultured at 37°C in a monolayer on 100 mm plates in MEM-10 medium (Gibco) containing 10% foetal calf serum (Reheis Chemical Co., Chicago, USA) in a water-saturated atmosphere containing 5% $CO_2$ in air. Prior to use, the cells were labelled with [$^{14}$C]thymidine (0.005 $\mu$Ci ml$^{-1}$, 55 mCi mol$^{-1}$) for 16 to 20 h. The medium was removed, and the plates were rinsed with PBS. Cells were trypsinized (0.025% trypsin in PBS), at 37°C for 10 min. A dilution of the suspension was counted by using a Coulter counter with appropriate corrections for coincidence.

### *Radioprotector*

2-[(Aminopropyl)amino] ethanethiol (WR-1065) was kindly supplied by Dr David E. Davidson, Jr., US Army Medical Research and Development Command, Fort Detrick, MD. For each experiment WR-1065 (Lot #BK-71365) was made up fresh at a concentration of 1 M in Dulbecco's PBS without calcium or magnesium (Gibco). The protector was routinely added to the selected cell suspensions to give a final concentration of 4 mM. This concentration was found to afford maximum protection to V79 cells with respect to radiation- or drug-induced cell killing and mutagenesis without evidence of any associated protector-induced toxicity (Grdina *et al.*, 1985*a*; Nagy *et al.*, 1986*a*).

### *Irradiation*

In dose-response experiments $5 \times 10^5$ cells, with or without protector, were placed in sterile, 15 ml centrifuge tubes and kept on ice until they were irradiated with a $^{60}$Co gamma irradiator (Gamma Beam 650: Atomic Energy of Canada) at a dose rate of 10 krad min$^{-1}$, with a total dose of from 2.5 to 25 krad (25–250 Gy). Immediately after irradiation, the suspension was diluted with ice-cold solution A (8 g NaCl, 0.4 g KCl, 1.0 g glucose, 0.35 g $NaHCO_3$ per liter) containing 5 mM EDTA to ensure inhibition of DNA repair (Meyn & Jenkins, 1983). In the DNA repair studies, a single cell suspension was irradiated, on ice, with a dose of 25 krad (250 Gy). The suspension was split into two fractions, which were placed in spinner flasks. To one was added sufficient protector to reach a final concentration of 4 mM, while the other served as the unprotected control. The flasks were incubated at 37°C. At 30, 60, 90, and 180 min, aliquots were removed and diluted with iced solution A with EDTA.

*Neutral elution*

The neutral elution procedure has been fully discussed elsewhere (Bradley & Kohn, 1979). Briefly, $5 \times 10^5$ cells were impinged onto a 25 mm diameter (0.8 $\mu$m pore size) polycarbonate filter (Nuclepore Corp., Pleasanton, CA, USA). Cells were washed once with 15 ml of solution A and lysed with 3 ml of a solution containing 0.05 M Tris, 0.05 M glycine, 0.025 M $Na_2EDTA$, and 2% (w/v) sodium lauryl sulphate. The pH was adjusted to 9.6 with Tris-base. Just prior to use, proteinase K was added (0.5 mg ml$^{-1}$; Sigma). This lysis solution was pumped through the filter unit for one hour at 2.13 ml h$^{-1}$, after which 50 ml of the lysis solution without proteinase K was added to the reservoir. The neutral elution solution was used at pH 9.6 as described by Bradley and Kohn (1979), or at pH 7.2 as suggested by Evans *et al.* (1986). Ninety-minute fractions were collected for 15 h at the same pump speed.

*Liquid scintillation counting*

The assay of DSB and their repair was accomplished by using liquid scintillation techniques. The filters were treated with 0.4 ml 1 N HCl for 1 h at 60°C. The filters were then cooled to room temperature and neutralized with 2.5 ml 0.4 M NaOH. All samples were counted in 15 ml cocktail consisting of 1 L toluene, 1 L Triton X-100 (Packard Inst. Co., Downers Grove, IL, USA) and 42 ml Liquiscint (ICN Chemical Corp., Irving, CA). A Beckman (LS2800) liquid scintillation spectrometer was used throughout. The data were presented as percent of [$^{14}$C]thymidine activity remaining on the filter as a function of elution volume.

*Strand scission factor calculation*

The designation of strand scission factor (SSF) refers to a relative value determined by comparison of associated DNA elution curves. This value is used to characterize relative numbers of DNA strand breaks. Specifically, SSF was determined from the relationship $SSF = |((fx)/(fo))|$, where $fo$ and $fx$ are, respectively, the proportions of DNA retained on the filter after volumes of 17.5 ml have been eluted for the nonirradiated control and the corresponding treated sample (Meyn & Jenkins, 1983).

*Relative strand scission factor calculation*

The relative SSF (RSSF) value is used to compare strand scission factors after allowing time between irradiation and assay for possible rejoining of DSB. It is the ratio of SSF values obtained in cells allowed no time for rejoining to those for cells allowed various amounts of time between irradiation and assay.

### Results

*Double-strand break formation*

The effect of the radiation protector WR-1065 (4 mM) on DSB formation was measured by using the neutral elution technique at pH 9.6 and pH 7.2. Figure 1 shows the dose response when the elution procedure was performed at pH 9.6. At each dose tested, DSB formation was reduced when the protector was present at the time of irradiation. The same experimental design with an elution solution at pH 7.2 caused a reduction in the detection of DSB formation for all doses tested (see Figure 3). A comparison of the two curves indicates that fewer DSB were observed when the procedure was run at the more neutral pH.

Figures 2 and 4 show the SSF values for elution procedures performed at pH 9.6 and pH 7.2, respectively. At

**Figure 1** Double strand break formation in V79 cells as determined by neutral elution at pH of 9.6. Dose response with and without 4 mM WR-1065 30 min prior to and during irradiation.

**Figure 2** Double strand scission factors (see text) for data presented in **Figure 1** at pH 9.6. Closed squares represent control V79 cells which had a slope of $0.307 \times 10^{-4}$. Closed circles represent WR-1065 treated cells which resulted in a slope of $0.213 \times 10^{-4}$. The dose modification factor is 1.44 for the data presented.

pH 9.6 unprotected V79 cells had a slope of $0.307 \times 10^{-4}$ while the protected cells had a slope of $0.213 \times 10^{-4}$. The reduced slope for the protected cells indicated the degree of protection afforded by WR-1065. The ratio of the two slopes reveals that WR-1065 reduced DSB formation by a factor of 1.44. Figure 4 shows similar results at pH 7.2. At this more neutral pH, the slopes for both protected and unprotected cells were less than corresponding slopes at pH 9.6. The slope for unprotected cells at pH 7.2 was $0.214 \times 10^{-4}$, while the slope for protected cells was $0.121 \times 10^{-4}$. The ratio of these slopes gives a protection factor of 1.77 for DSB formation at the lower pH.

**Figure 3** Double strand break formation in V79 cells as determined by neutral elution at pH of 7.2. Dose response with and without 4 mM WR-1065 30 min prior to and during irradiation.

**Figure 5** Neutral elution patterns of unprotected V79 cells allowed 0, 30, 60, 90 or 180 min between irradiation (250 Gy) and assay. The eluting solution had a pH of 9.6.

**Figure 4** Double strand scission factors (see text) for data presented in **Figure 2** at pH 7.2. Closed squares represent control V79 cells which had a slope of $0.214 \times 10^{-4}$. Closed circles represent WR-1065 treated cells which resulted in a slope of $0.121 \times 10^{-4}$. The dose modification factor is 1.77 for the data presented.

**Figure 6** Neutral elution patterns of V79 cells treated with 4 mM WR-1065 for 0, 30, 60, 90 or 180 min between irradiation (250 Gy) and assay. Crossed circles designate unirradiated control cells exposed to protector for similar time. The eluting solution had a pH of 9.6.

*Rejoining of double-strand breaks*

Figures 5 and 6 show the effect of the protector (Figure 6) on the elution kinetics of rejoining of DSB at pH 9.6 after exposure to a dose of 25 krad. In these experiments, V79 cells were irradiated without WR-1065 and then allowed time to repair at 37°C either in the presence or absence of protector. Values of relative strand scission factor (RSSF) were plotted against repair time at pH 9.6 for protected and unprotected V79 cells (Figure 7). These procedures, detected no effect of the protector on rejoining of DSB. The results obtained when the elution solution had a pH of 7.2 are shown in Figures 8–10; they are similar to results at pH 9.6.

At pH 9.6, the half-life of rejoining was approximately 184 min in both the protected and unprotected V79 cells. The biphasic nature of the RSSF curve seen in Figure 7, however, suggests that heterogeneity exists; this may indicate that both SSB and DSB were measured by the assay procedure. At pH 7.2, the half-life of repair was ~102 min in both groups. Qualitatively, the curve describing the kinetics

**Figure 7** Relative strand scission factors (see text) in protected and unprotected V79 cells allowed varying times for repair with subsequent elution at pH 9.6.

**Figure 9** Neutral elution patterns of V79 cells treated with 4 mM WR-1065 for 0, 30, 60, 90 or 180 min between irradiation (250 Gy) and assay. Crossed open circle designate unirradiated controls exposed to protector for a similar period. The eluting solution had a pH of 7.2.

**Figure 8** Neutral elution patterns of unprotected V79 cells allowed 0, 30, 60, 90 or 180 min between irradiation (250 Gy) and assay. The eluting solution had a pH of 7.2.

**Figure 10** Relative strand scission factors in protected and unprotected V79 cells allowed varying times for repair with subsequent elution at pH 7.2.

of repair of DSB at pH 9.6 appears to be more heterogeneous than do the exponential repair kinetics seen (Figure 10) when the elution solution was at pH 7.2.

### Discussion

Published studies have described non-traditional uses of chemical radiation protectors such as modulating the mutagenic (Zwelling *et al.*, 1979; Bradley *et al.*, 1982; Shrieve & Harris, 1982; Nagy *et al.*, 1986*b*) and carcinogenic (Milas *et al.*, 1984; Hill *et al.*, 1986) effects of treatment with either radiation or anti-cancer agents. Because the mechanism of action of these agents apparently involves gross genetic damage, initial studies described the role of radiation protectors on single-strand breaks at biologically relevant radiation doses (Grdina & Nagy, 1986).

The present investigation extends these studies, and describes the protective effects of WR-1065 on the radiation induced double-strand DNA breaks and repair using the neutral elution assay (Bradley & Kohn, 1979). This procedure is believed to correlate better with radiation induced cell killing (Hutchinson, 1978; Radford, 1985) but is relatively insensitive and requires extremely high, supra-lethal radiation doses. Recent reports (Tilby, 1984; Evans *et al.*, 1986) have expressed concern that the pH originally suggested for the neutral elution procedure (pH 9.6) may include some single-stranded DNA. It was for this reason that the experiments reported here used neutral elution buffers at a pH of 7.2 and compared the results with the elution procedure using buffers at the conventional pH of 9.6. Our results indicate that the assay when performed at pH 7.2 resulted in the detection of fewer DNA breaks than at a pH of 9.6. The reasons for this difference are unknown but may involve the inclusion of measurable SSB at pH 9.6 and/or the higher pH may result in an increase in DSB formation due to hydrolysis of alkali-labile bonds in the damaged DNA (Tilby *et al.*, 1984).

The presence of WR-1065 during irradiation reduces the frequency of DSB formation detected at either pH. The linear dose response relationship for strand-scission factors indicates a quality of DNA damage which is directly proportional to dose at either of the pH's tested. These results are similar to those reported (Grdina & Nagy, 1986) previously for SSB using the alkaline elution technique.

The presence of protector during repair with subsequent elution at the two pH's tested, shows that it does not modify the DSB repair kinetics at either pH. Grdina and Nagy (1986) found that WR-1065 inhibited rejoining of radiation induced SSB. Further, they found that the protector inhibited progression in the cell cycle, which may have allowed the cells more time for repair prior to cell division. This in turn, could lead to an enhanced fidelity of repair which would be reflected in the reduction of SSB formation and reduced mutation frequency (Grdina *et al.*, 1985*a*). The results reported here, using very high radiation doses, indicate that double-strand rejoining is still incomplete at 180 min whether protector was present or not and at either pH. The relative rates of rejoining, as determined by neutral elution kinetics, were apparently affected by changing the pH of the elution buffer. At pH 9.6 the kinetics appeared to be bi-phasic while at pH 7.2 the repair kinetics were exponential.

The differences noted between the effects of WR-1065 on rejoining of DNA SSB and DSB is not surprising because there is no *a priori* reason to assume that similar mechanisms are involved. The lack of a template in DSB rejoining presents problems not seen in SSB repair. In addition, the extremely high radiation doses required for neutral elution could possibly be inducing damage which involve more and different targets than is seen with lower doses evaluated using the alkaline elution method.

It has been suggested that WR-1065 can react rapidly with oxygen in the culture medium, leading to a state of transient hypoxia (Purdie *et al.*, 1983; Durand, 1983; Biaglow *et al.*, 1984). This could partially explain the reduction in DSB DNA damage reported in the present study. However, the protective effect observed against radiation induced mutagenesis at the HGPRT locus in V79 cells has been observed even under conditions of acute hypoxia (Nagy *et al.*, 1986*b*). Since mutation induction is presumably due to genetic damage, it would appear that the mechanism of WR-1065 protection against radiation induced DSB formation can not be explained solely by an oxygen depletion mechanism.

The results reported here show quantitative differences depending upon the pH of the neutral elution buffers used, but the qualitative response with WR-1065 present is the same at either pH. Therefore, the conclusions drawn are similar regardless of which pH is used. WR-1065 is effective in reducing DSB formation in irradiated V79 cells and it apparently does not interfere with rejoining of DSB. The heterogeneity noted in the relative strand scission factors at pH 9.6 may suggest the possibility of inclusion of significant numbers of SSB in the neutral elution assay. Regardless, it is clear that this class of protectors can reduce radiation induced DNA damage if present during irradiation.

Additional studies are obviously required to better understand the interactions of this class of protectors with radiation induced DNA damage and repair. Information of this type will be useful in the expanding applications of protectors in cancer treatment and its prevention.

The authors acknowledge the expert technical assistance of Ms Phylis Dale and Ms Jane Angerman. We are indebted to G. Holmblad, for dosimetry determinations and to Ms C. Fox for computer analysis of the data.

The protector used throughout these experiments was kindly provided by COL David E. Davidson, Jr., Director, Division of Experimental Therapeutics, Walter Reed Army Medical Center, Washington, D.C. 20307, USA.

The research was funded in part by the US Department of Energy under contract No. W-31-109-ENG-38 and DHHS, NIH/NCI grant No. CA-37435. The first author (CPS) is indebted to Argonne National Laboratory, the Division of Biological and Medical Research and the Division of Educational Programs for partial support of the sabbatical.

### References

BIAGLOW, J.E., ISSELS, R.W., GERWECK, L.E. & 4 others (1984). Factors influencing the oxidation of cysteamine and other thiols: Implications for hyperthemic sensitization and radiation protection. *Radiat. Res.*, **100**, 298.

BRADLEY, M.O. & KOHN, K.W. (1979). X-ray induced DNA double strand break production and repair in mammalian cells as measured by neutral filter elution. *Nucleic Acid Res.*, **7**, 793.

BRADLEY, M.O., PATTERSON, S. & ZWELLING, L.A. (1982). Thiourea prevents cytotoxicity and mutagenicity, but not sister-chromatid exchanges in V79 cells treated with *cis*-diammine-dichloroplatinum (II). *Mutat. Res.*, **96**, 67.

DURAND, R.E. (1983). Radioprotection by WR-2721 *in vitro* at low oxygen tensions: implication for its mechanisms of action. *Br. J. Cancer*, **47**, 387.

EVANS, J.W., LIMOLI, C.L. & WARD, J.F. (1986). Does neutral elution measure intracellular levels of DNA double strand breaks (DSB)? *34th Annual Meeting of the Radiation Research Society*, Las Vegas, (*Abstract Cq-17*).

GRDINA, D.J. & NAGY, B. (1986). The effect of WR-1065 on radiation induced DNA damage and repair and cell progression in V79 cells. *Br. J. Cancer*, **54**, 933.

GRDINA, D.J., NAGY, B., HILL, C.K., WELLS, R.L. & PERAINO, C. (1985*a*). The radioprotector WR-1065 reduces radiation-induced mutations at the hypoxanthine-guanine phosphoribiosyl transferase locus in V79 cells. *Carcinogenesis*, **6**, 929.

GRDINA, D.J., PERAINO, C., CARNES, B.A. & HILL, C.K. (1985*b*). Protective effect of S-2-(3-aminopropylamino) ethylphosphorothioic acid against induction of altered hepatocyte foci in rats treated once with gamma radiation within one day after birth. *Cancer Res.*, **45**, 5379.

HILL, C.K., NAGY, B., PERAINO, C. & GRDINA, D.J. (1986). WR-1065 is anti-neoplastic and anti-mutagenic when given during Co-60 gamma irradiation. *Carcinogenesis*, **7**, 665.

HUTCHINSON, F. (1978). DNA strand break repair in eukaryotes. In *DNA Repair Mechanisms*. Hanawalt *et al.*, (eds) p. 457, Academic Press: New York, Workshop summary.

KLIGERMAN, M.M., SHAW, M.T., SLAVID, M., YUHAS, J.M. (1980). Phase I clinical studies with WR-2721. *Cancer Clin. Trials*, **3**, 217.

KLIGERMAN, M.M., GLOVER, D.J., TURRISI, A.T. & 6 others (1984). Toxicity of WR-2721 administered in single and multiple doses. *Int. J. Radiat. Oncol. Biol. Phys.*, **10**, 1773.

MEYN, R.E. & JENKINS, W.T. (1983). Variation in normal and tumor tissue sensitivity of mice to ionizing radiation-induced DNA strand breaks *in vivo*. *Cancer Res.*, **43**, 5668.

MILAS, L., HUNTER, N., STEPHENS, C.L. & PETERS, L.J. (1984). Inhibition of radiation carcinogenesis by WR-2721. *Cancer Res.*, **44**, 5567.

NAGY, B., DALE, P.J. & GRDINA, D.J. (1986*a*). Protection against *cis*-diamminedichloroplatinum cytotoxicity and mutagenicity in V79 cells by 2-[(aminopropyl) amino] ethanethiol. *Cancer Res.*, **45**, 1132.

NAGY, B., HILL, C.K. & GRDINA, D.J. (1986*b*). Protective effects of WR-1065 even under conditions of acute hypoxia, against radiation induced mutagenicity in V-79 cells. *Pro. Amer. Assoc. Cancer Res.*, **27**, 150.

PURDIE, J.W., INHABER, E.R., SCHNEIDER, H. & LABELLE, J.L. (1983). Interaction of cultured mammalian cells with WR-2721 and its thiol, WR-1065: Implications for mechanisms of radioprotection. *Int. J. Radiat. Biol.*, **43**, 517.

RADFORD, I.R., (1985). The level of induced DNA double-strand breakage correlates with cell killing after x-irradiation. *Int. J. Radiat. Biol.*, **48**, 45.

SHRIEVE, D.C. & HARRIS, J.W. (1982). Protection against *cis*-dichlorodiammine Pt (II) cytotoxicity *in vitro* by cysteamine. *Int. J. Radiat. Oncol. Biol. Phys.*, **8**, 585.

TAKAHASI, I., NAGAI, T., MIYAISHI, K., MAEHARA, Y. & NIIBE, H. (1986). Clinical study of the radioprotective effects of Amifostine (YM-08310, WR-2721) on chronic radiation injury. *Int. J. Radiation Oncology Biol. Phys.*, **12**, 935.

TILBY, M.J., LOVEROCK, P.S. & FIELDEN, E.M. (1984). An effect of elevated postirradiation pH on the yield of double-strand breaks in DNA from irradiated bacterial cells. *Radiat. Res.*, **98**, 284.

UTLEY, J.F., MARLOWE, C. & WADDELL, W.J. (1976). Distribution of 35-S labelled WR-2721 in normal and malignant tissues of the mouse. *Radiat. Res.*, **68**, 284.

WARD, J. (1986). Mechanisms of DNA repair and their potential modification for radiotherapy. *Int. J. Radiat. Oncol. Biol. Phys.*, **12**, 1027.

YUHAS, J.M. & STORER, J.B. (1969). Differential chemoprotection of normal and malignant tissues. *J. Natl Cancer Inst.*, **42**, 331.

YUHAS, J.M., SPELLMAN, J.M. & CULO, F. (1980). The role of WR-2721 in radiotherapy and/or chemotherapy. *Cancer Clin. Trials*, **3**, 211.

YUHAS, J.M. (1980). A more general role for WR-2721 in cancer therapy. *Br. J. Cancer*, **41**, 832.

ZWELLING, L.A., FILIPSKI, J. & KOHN, K.W. (1979). Effect of thiourea on survival and DNA cross-link formation in cells treated with platinum (II) complexes, L-phenylalanine mustard, and bis(2-chloroethyl) methylamine. *Cancer Res.*, **39**, 4989.


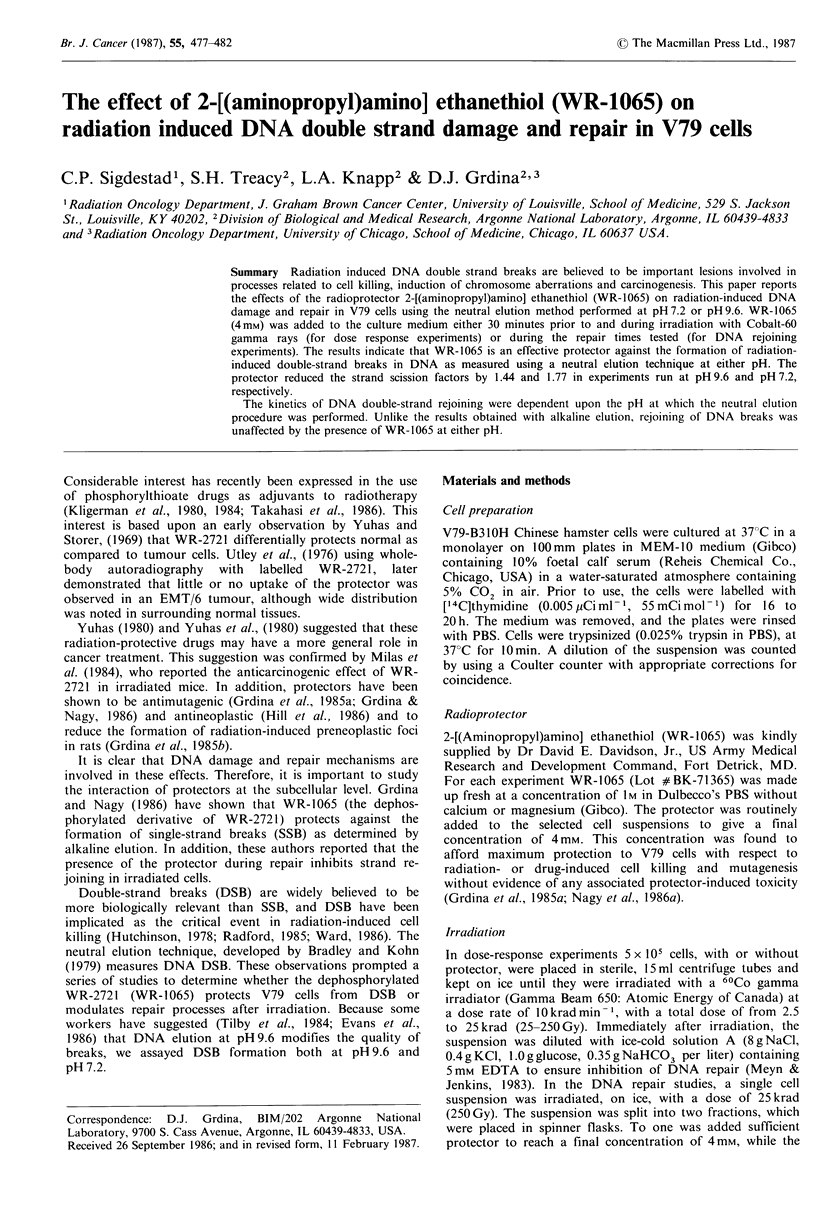

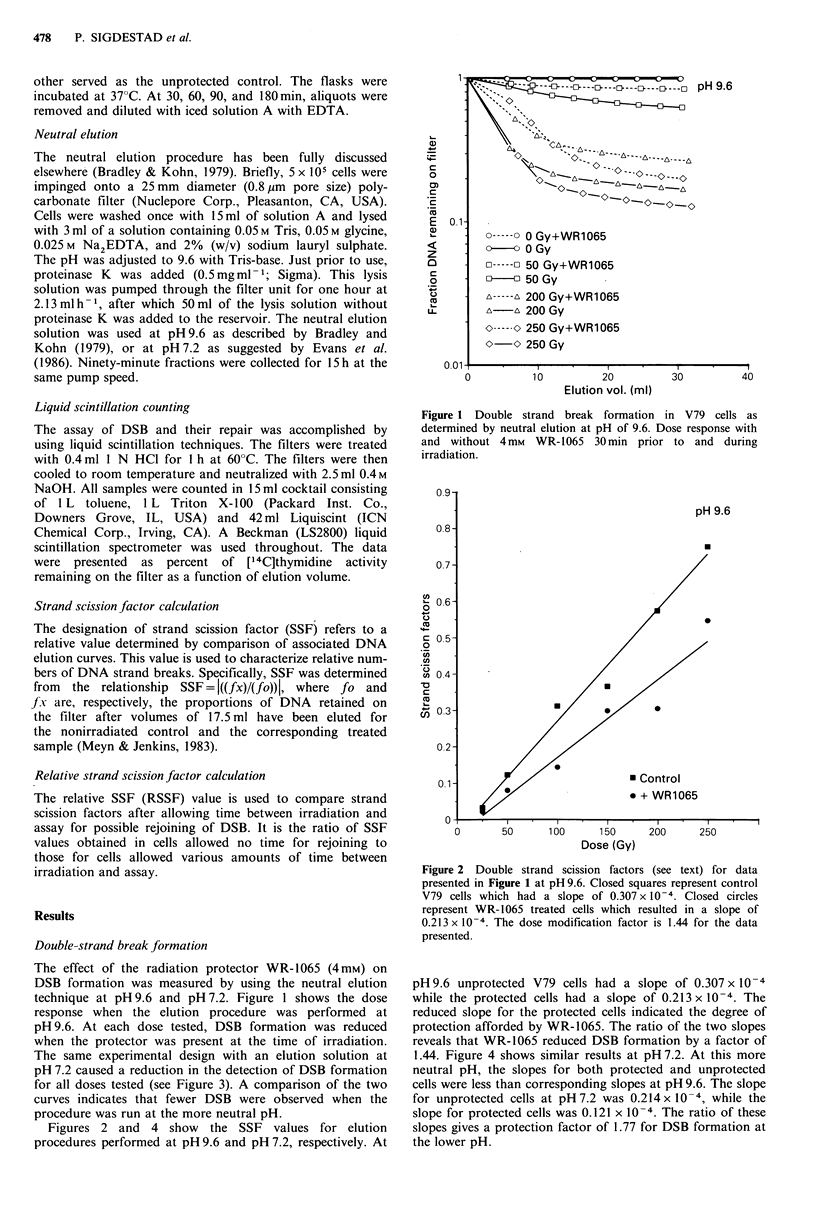

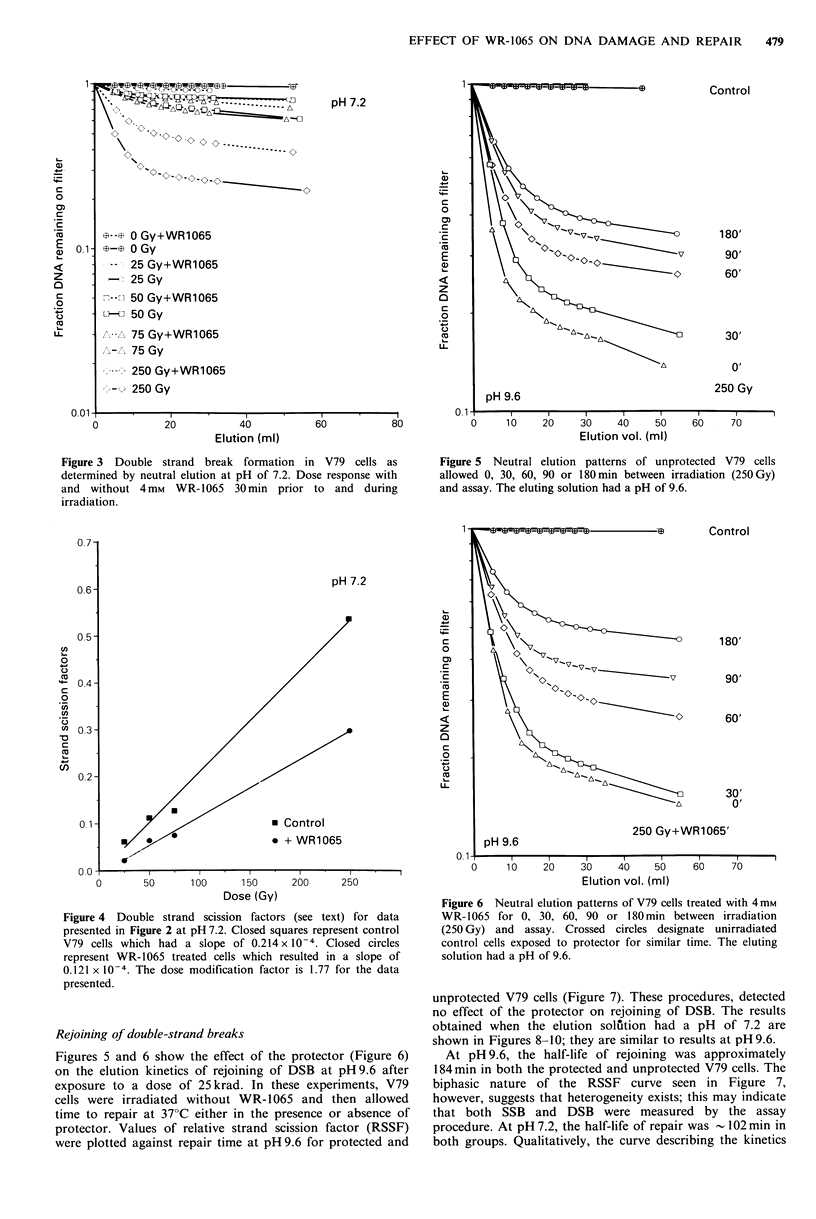

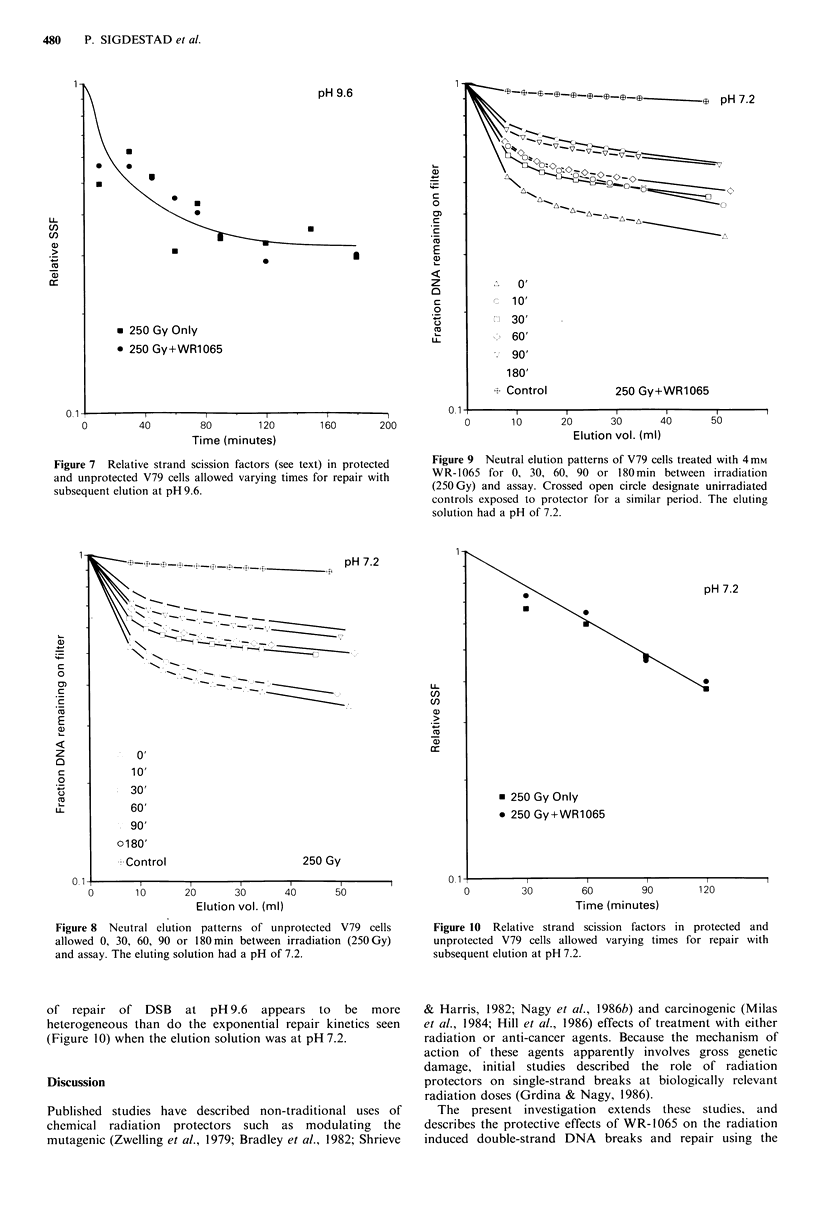

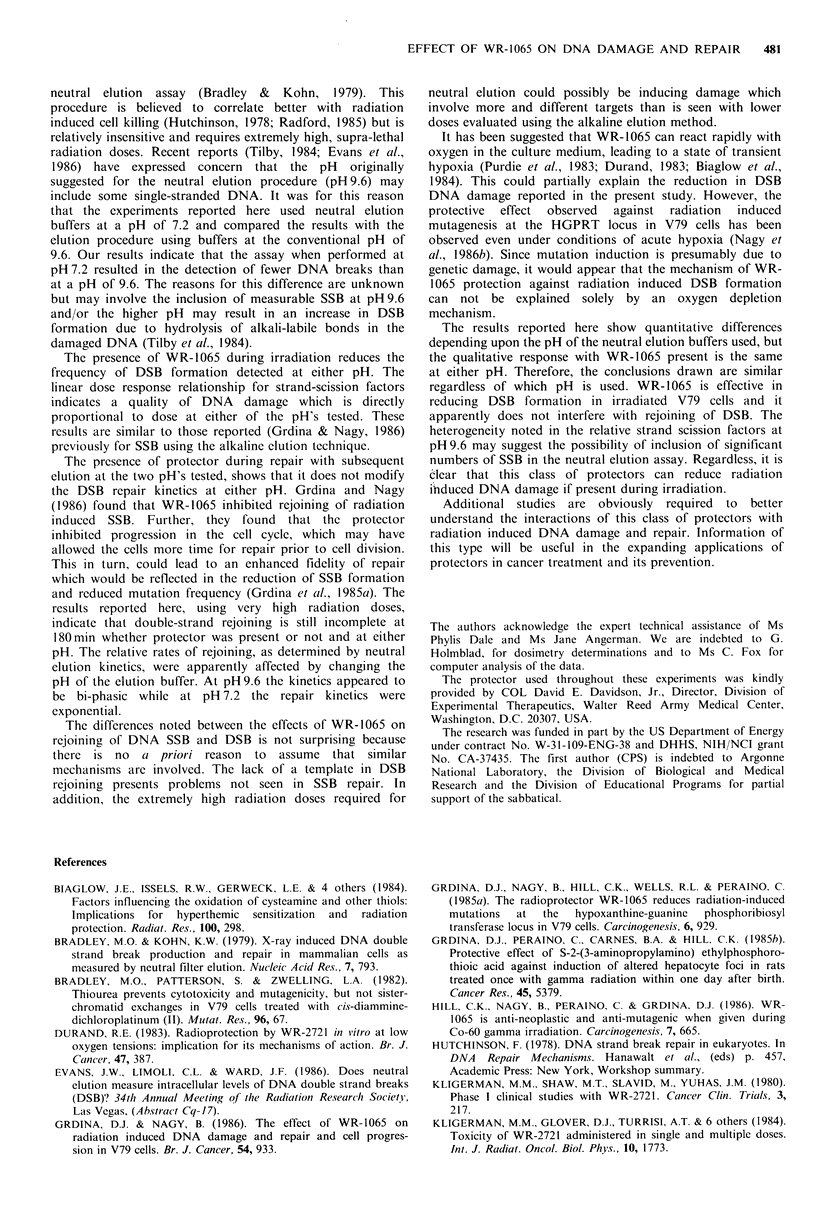

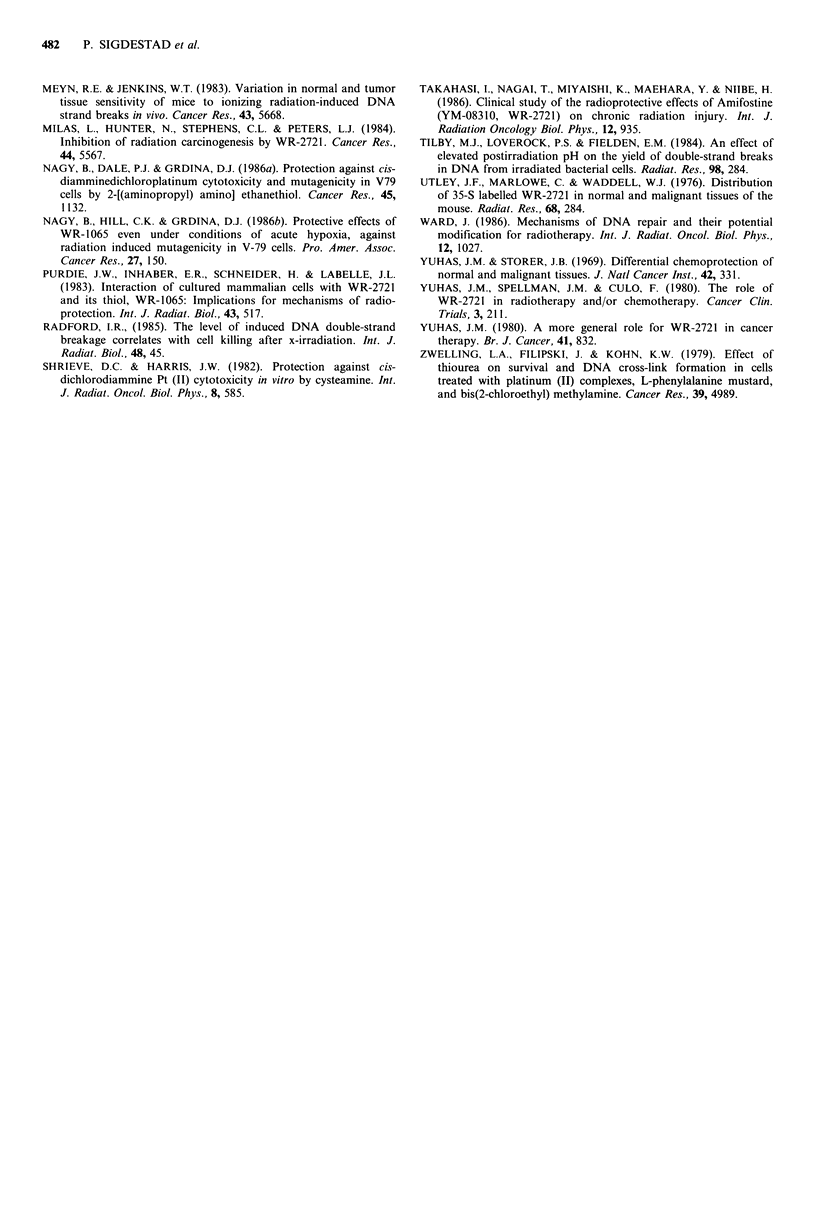


## References

[OCR_00858] Biaglow J. E., Issels R. W., Gerweck L. E., Varnes M. E., Jacobson B., Mitchell J. B., Russo A. (1984). Factors influencing the oxidation of cysteamine and other thiols: implications for hyperthermic sensitization and radiation protection.. Radiat Res.

[OCR_00864] Bradley M. O., Kohn K. W. (1979). X-ray induced DNA double strand break production and repair in mammalian cells as measured by neutral filter elution.. Nucleic Acids Res.

[OCR_00869] Bradley M. O., Patterson S., Zwelling L. A. (1982). Thiourea prevents cytotoxicity and mutagenicity, but not sister-chromated exchanges in V79 cells treated with cis-diaminedichloroplatinum (II).. Mutat Res.

[OCR_00875] Durand R. E. (1983). Radioprotection by WR-2721 in vitro at low oxygen tensions: implications for its mechanisms of action.. Br J Cancer.

[OCR_00891] Grdina D. J., Nagy B., Hill C. K., Wells R. L., Peraino C. (1985). The radioprotector WR1065 reduces radiation-induced mutations at the hypoxanthine-guanine phosphoribosyl transferase locus in V79 cells.. Carcinogenesis.

[OCR_00886] Grdina D. J., Nagy B. (1986). The effect of 2-[(aminopropyl)amino] ethanethiol (WR1065) on radiation-induced DNA damage and repair and cell progression in V79 cells.. Br J Cancer.

[OCR_00904] Hill C. K., Nagy B., Peraino C., Grdina D. J. (1986). 2-[(Aminopropyl)amino]ethanethiol (WR1065) is anti-neoplastic and anti-mutagenic when given during 60Co gamma-ray irradiation.. Carcinogenesis.

[OCR_00919] Kligerman M. M., Glover D. J., Turrisi A. T., Norfleet A. L., Yuhas J. M., Coia L. R., Simone C., Glick J. H., Goodman R. L. (1984). Toxicity of WR-2721 administered in single and multiple doses.. Int J Radiat Oncol Biol Phys.

[OCR_00914] Kligerman M. M., Shaw M. T., Slavik M., Yuhas J. M. (1980). Phase I clinical studies with WR-2721.. Cancer Clin Trials.

[OCR_00926] Meyn R. E., Jenkins W. T. (1983). Variation in normal and tumor tissue sensitivity of mice to ionizing radiation-induced DNA strand breaks in vivo.. Cancer Res.

[OCR_00931] Milas L., Hunter N., Stephens L. C., Peters L. J. (1984). Inhibition of radiation carcinogenesis in mice by S-2-(3-aminopropylamino)-ethylphosphorothioic acid.. Cancer Res.

[OCR_00936] Nagy B., Dale P. J., Grdina D. J. (1986). Protection against cis-diamminedichloroplatinum cytotoxicity and mutagenicity in V79 cells by 2-[(aminopropyl)amino]ethanethiol.. Cancer Res.

[OCR_00948] Purdie J. W., Inhaber E. R., Schneider H., Labelle J. L. (1983). Interaction of cultured mammalian cells with WR-2721 and its thiol, WR-1065: implications for mechanisms of radioprotection.. Int J Radiat Biol Relat Stud Phys Chem Med.

[OCR_00959] Shrieve D. C., Harris J. W. (1982). Protection against cis-dichlorodiammine Pt (II) cytotoxicity in vitro by cysteamine.. Int J Radiat Oncol Biol Phys.

[OCR_00964] Takahashi I., Nagai T., Miyaishi K., Maehara Y., Niibe H. (1986). Clinical study of the radioprotective effects of Amifostine (YM-08310, WR-2721) on chronic radiation injury.. Int J Radiat Oncol Biol Phys.

[OCR_00970] Tilby M. J., Loverock P. S., Fielden E. M. (1984). An effect of elevated postirradiation pH on the yield of double-strand breaks in DNA from irradiated bacterial cells.. Radiat Res.

[OCR_00975] Utley J. F., Marlowe C., Waddell W. J. (1976). Distribution of 35S-labeled WR-2721 in normal and malignant tissues of the mouse1,2.. Radiat Res.

[OCR_00980] Ward J. F. (1986). Mechanisms of DNA repair and their potential modification for radiotherapy.. Int J Radiat Oncol Biol Phys.

[OCR_00994] Yuhas J. M. (1980). A more general role for WR-2721 in cancer therapy.. Br J Cancer.

[OCR_00989] Yuhas J. M., Spellman J. M., Culo F. (1980). The role of WR-2721 in radiotherapy and/or chemotherapy.. Cancer Clin Trials.

[OCR_00985] Yuhas J. M., Storer J. B. (1969). Differential chemoprotection of normal and malignant tissues.. J Natl Cancer Inst.

[OCR_00998] Zwelling L. A., Filipski J., Kohn K. W. (1979). Effect of thiourea on survival and DNA cross-link formation in cells treated with platinum(II) complexes, L-phenylalanine mustard, and bis(2-chloroethyl)methylamine.. Cancer Res.

